# Application of Simple Imaging Technique for Fluorescence Bronchoscope

**DOI:** 10.1155/DTE.1.79

**Published:** 1994

**Authors:** Harubumi Kato, Tetsuya Okunaka, Norihiko Ikeda, Chimori Konaka

**Affiliations:** Department of Surgery Tokyo Medical College Hospital 6-7-1 Shinjuku-ku Nishishinjuku Tokyo 160 Japan

## Abstract

It was reported that the significant spectral difference of autofluroescence induced by laser light between cancer and normal tissue. A fluorescence bronchoscope system with simple light source and light filter was newly developed. In this paper, the detection of autofluorescence from bronchogenic dysplasia with this system was reported.


*Diagnostic and Therapeutic Endoscopy*, Vol. 1, pp. 79–81
Reprints available directly from the publisher
Photocopying permitted by license only

© 1994 Harwood Academic Publishers GmbH
Printed in Malaysia

### Application of Simple Imaging Technique for Fluorescence Bronchoscope: Preliminary Report

HARUBUMI KATO, TETSUYA OKUNAKA, NORIHIKO IKEDA, and CHIMORI KONAKA

*Department of Surgery, Tokyo Medical College Hospital, 6-7-1 Shinjuku-ku, Nishishinjuku, Tokyo 160 Japan*

*(Received July 6, 1994; in final form July 7, 1994)*

It was reported that the significant spectral difference of autofluroescence induced by laser light between cancer and normal tissue. A fluorescence bronchoscope system with simple light source and light filter was newly developed. In this paper, the detection of autofluorescence from bronchogenic dysplasia with this system was reported.

**KEY WORDS:** fluorescence bronchoscope, bronchogenic carcinoma in situ

### INTRODUCTION

Detection of carcinoma in situ is a diagnostic challenge even for the experienced bronchoscopists. These lesions do not always show mucosal changes visible by conventional bronchoscopy (Woolner *et al.*, 1984). Several attempts were made in the field of photodynamic diagnosis to enhance and localize such lesions more specifically (Hayata *et al.*, 1993; Kato *et al.*, 1990). However, this modality requires photosensitizer, which has some side effect and is not easily available (Lam *et al.*, 1991). To overcome this problem, only low-dose photosensitizers were used (Lam *et al.*, 1991), and also several endoscopic fluorescence detection systems have been created (Lam *et al.*, 1991; Profio *et al.*, 1984; Lam *et al.*, 1993). Laser light was used for the excitation of autofluorescence, and sophisticated techniques also were necessary to amplify the fluorescence signal in all devices (Lam *et al.*, 1991; Profio *et al.*, 1984; Lam *et al.*, 1993).

The authors tried to simplify the system and invented a new system with a conventional Xenon lamp excitation and image intensifier. Diagnostic images were obtained using this device in dysplasia lesions that could not be detected by conventional brochoscopy. This is a preliminary report of a newly developed endoscope for detection of tissue/mucosal autofluorescence without employing a laser.

Address correspondence to: Dr. Harubumi Kato, M.D., Ph.D., Department of Surgery, Tokyo Medical College, 6-7-1 Shinjuku-ku, Nishishinjuku, Tokyo, 160 Japan.

### MATERIALS AND METHODS

### Fluorescence Endoscope

A Xe-lamp was used as the excitation light source (Fig. 1). Infrared light was eliminated by the infrared cut filter (IR cut filter), and 420 to 480 nm exitation light was delivered through an excitation filter (EX Filter) and transmitted to the target via a light guide (Fig. 2A). The emitted fluorescence was collected and transmitted via an image guide. The camera, which contained a fluorescence filter (FL filter), image intensifier, and a TV camera was attached to the eyepiece of the bronchoscope. Only 520 to 600 nm light passed through the FL filter and amplified by image intensifier (Fig. 2B).

The intensified autofluorescence of the normal mucosa appeared green in the image monito, although the abnormal area showed a cold image caused by the lack of autofluorescence. The images can be processed in real time

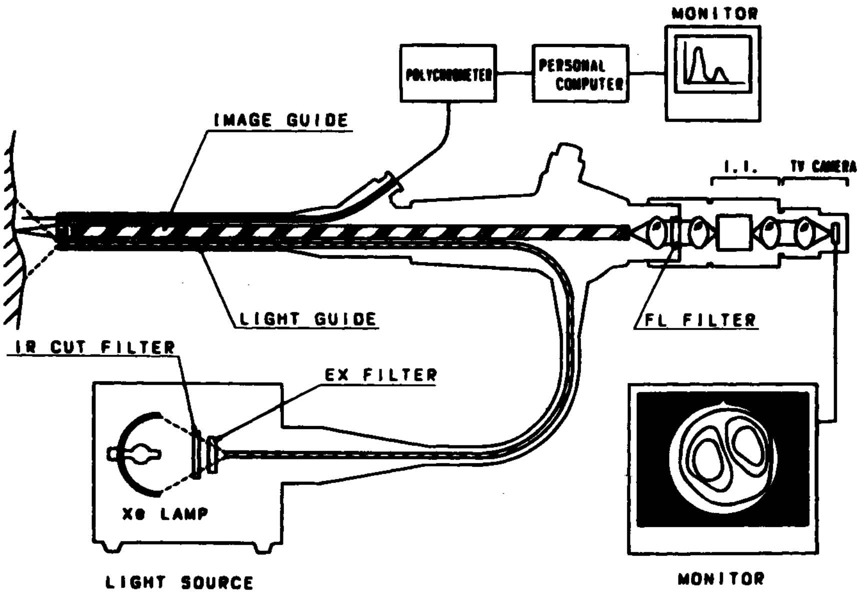

**Figure 1** Xenon lamp fluorescence endoscope system. Only 420–480-nm excitation light is passed by the IR cutting filter and transmitted to the target through an EX filter. The autofluorescence is transmitted by the image guide of the bronchoscope, and only 520–600 nm emitted light was passed by the FL filter.

and the fluorescence image displayed simultaneously on the video monitor as a color image.

### Subject

Moderately dysplastic cells were obtained by sputum cytology in a 62 year-old-male who underwent a general check-up and was referred to our clinic. Chest x-ray findings were negative, and blood test results were all normal. Endoscopic examination was scheduled to localize the disease.

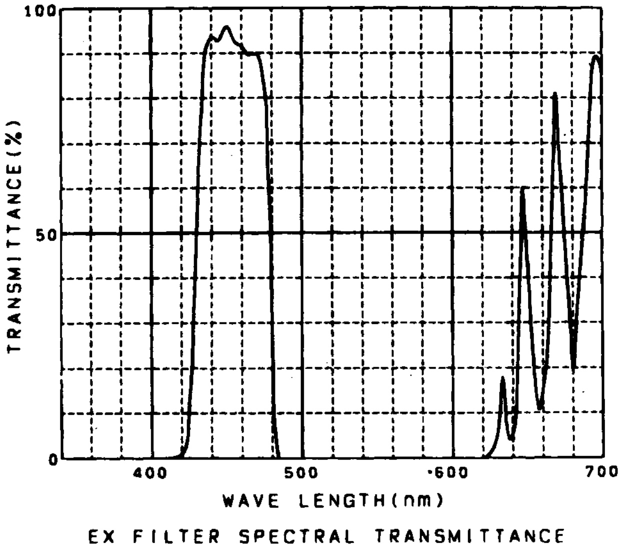

**Figure 2A** Transmission rate of the EX filter, showing it to pass only 420–480 nm light.

### RESULTS

Figure 3 shows the images of the left upper lobe by conventional bronchoscopy and fluorescence endoscope. No abnormal findings were recognized by conventional brochoscopy. Using our endoscopic autofluorescence system, a cold spot was located in the membranous portion in the orifice of the left upper lobe. Brushing cytology of the cold spot showed moderate dysplasia. No other abnormal finding was detected by either method.

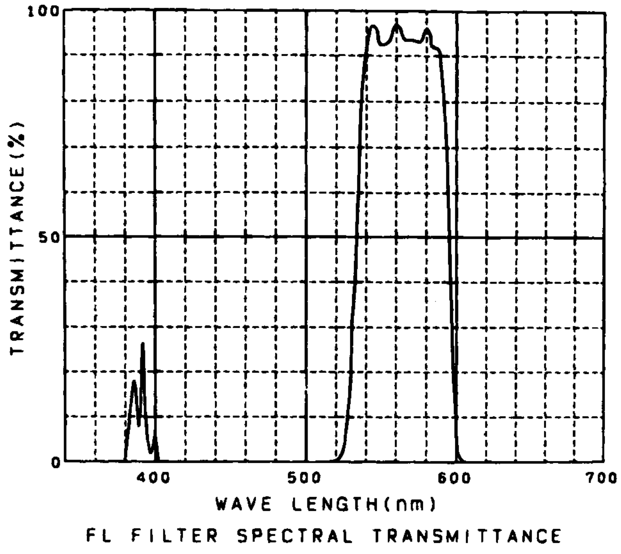

**Figure 2B** The FL filter passes only 520–600 nm light.

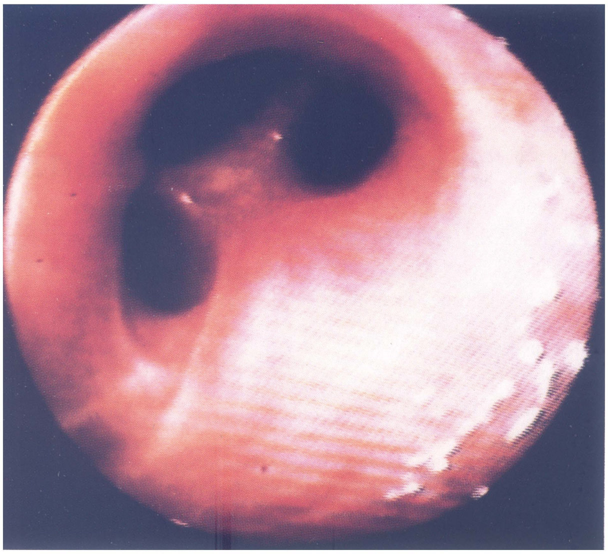

**Figure 3A** Appearance of the left upper lobe by conventional bronchoscopy. No abnormal finding was detected.

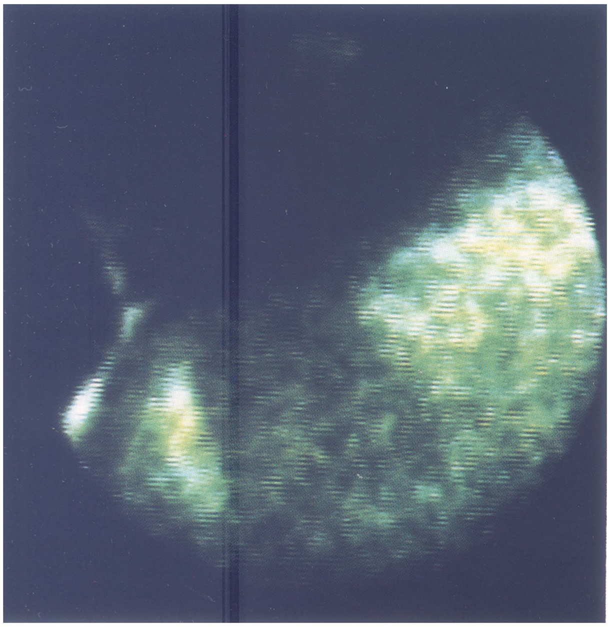

**Figure 3B** Image of the same site seen through the fluorescence endoscopy system. Normal tissue appeared green, and the site of dysplasia appeared dark brown.

### DISCUSSION

Dysplasia and carcinoma in situ are difficult to detect by conventional bronchoscopy, because these lesions may not always show enough macroscopically recognizable changes in the bronchial mucosa. This fact was supported by the report of Woolner *et al.* (Woolner *et al.*, 1984). To overcome this problem, clinical trials using various photosensitizers have been performed for many years, and better sensitivity for early lung cancer was obtained (Furuse *et al.*, 1993; Hayata *et al.*, 1993; Kato *et al*, 1990). However, photosensitizers generally produce skin photosensitivity, which makes these drugs less suitable for routine examinations.

Recently, imaging devices employing tissue autofluorescence have been developed (Lam *et al.*, 1991; Profio *et al.*, 1984). Lam *et al.* reported the significant spectral difference of autofluorescence induced by laser light between cancer and normal tissue. They have used their system for the detection of early cancer as well as of dysplasia (Lam *et al.*, 1993). The system described in this report is the first clinical prototype of a fluorescence endoscope with a simple (i.e., nonlaser) light source (Xe-lamp) and light filter. Further analyses on spectra and effects of distance, angle of incidence, and tissue reflective properties are necessary for clinical applications. Autofluorescence endoscopic system will become more simple and easier after this system becomes more sophisticated.

Fluorescence endoscopy is as easy to perform as the conventional bronchoscopic procedure, and it holds great promise in the detection of changes of cancerous/precancerous lesions not recognizable by the human eye with conventional endoscopes.

### ACKNOWLEDGEMENTS

The authors thank Asahi Optical Co. Ltd. and Hamamatsu Photonics Co. Ltd. for their exellent technical support in the development of the system.

### REFERENCES

Furuse K., Fukuoka M., Kato H., *et al.* (1993) A prospective study on photodynamic therapy with Photofrin II for centrally located early-stage lung cancer. *J Clin Oncol* 11:1852–1857.

Hayata Y., Kato H., Konaka C., *et al.* (1993) Photodynamic therapy in early stage lung cancer. Lung Cancer; 9:287–294.

Kato H., Imaizumi T., Aizawa K., *et al.* (1990) Photodynamic diagnosis in respiratory tract malignancy using an eximer dye laser system. *J Photochem Photobiol* 6:189–196.

Lam S., Hung J., Palcic B. (1991) Mechanism of detection of early lung cancer by ratio fluorometry. Laser in the Life Science 4:67–73.

Lam S., MacAulay C., Hung J., *et al.* (1993) Detection of dysplasia and carcinoma in situ with a lung imaging fluorescence endoscope device. *J Thorac Cardiovasc Surg* 105:1035–1040.

Profio E., Doiron R., Sarnaik J. (1984) Fluorometer for endoscopic diagnosis of tumors. *Med Phys* 11:516–535.

Woolner L. B., Fontana R. S., Cortese D. A., *et al.* (1984) Roentgegraphically occult lung cancer: pathologic findings and frequency of multicentricity during a 10-year period. *Mayo Clin Proc* 59:453–466.

